# Low-Cost Preparation of Hydrophobic Silica Aerogels from Water Glass Using Water as the Sole Solvent

**DOI:** 10.3390/ma19153313

**Published:** 2026-08-04

**Authors:** Pengzhai Li, Kangzhen Sun, Yi Wu, Qiuli Fang, Yin Zhang

**Affiliations:** College of Materials Science and Engineering, Nanjing Tech University, Nanjing 211816, China; 202361203204@njtech.edu.cn (P.L.);

**Keywords:** freeze-drying, vapor deposition, sodium silicate, sol–gel, silica aerogels

## Abstract

**Highlights:**

- A low-cost silica aerogel route is developed using water as the sole solvent.
- Controlled gelation enables the formation of a uniform mesoporous network.
- Vapor-phase silylation converts the aerogel surface from hydrophilic to hydrophobic.
- The resulting aerogels show promising thermal insulation performance and potential for green scalable production.

**Abstract:**

To address the dependence on organic solvents, costly silicon precursors, and complex processing in conventional silica aerogel preparation, this study developed a green and low-cost aqueous route using water glass as the silicon source. The sol–gel process was optimized through an orthogonal experimental design by regulating precursor concentration, pH, temperature, and catalyst dosage, enabling the formation of a stable three-dimensional silica network. Under the optimized conditions, the unmodified silica aerogel exhibited low density, high porosity, and a typical mesoporous structure, with a specific surface area of 707.87 m^2^/g, an average pore size of 5.75 nm, and a thermal conductivity of 0.0408 W/(m·K). After HMDS vapor-phase modification, hydrophobic methyl groups were introduced onto the aerogel surface, increasing the water contact angle to 132.3°. Among the modified samples, S3 showed the lowest thermal conductivity of 0.0360 W/(m·K), indicating good thermal insulation performance. This work provides a feasible strategy for preparing hydrophobic silica aerogels through a cost-effective aqueous process, showing potential for greener and large-scale production of silica aerogel materials.

## 1. Introduction

Silica aerogel is a lightweight, highly porous nanomaterial characterized by a distinctive three-dimensional network structure, and has shown great potential for applications in areas such as building energy conservation, industrial catalysis, environmental adsorption, and aerospace and military industries due to its ultra-high porosity (typically >90%), extremely low density, and excellent thermal insulation properties [1,2,3,4]. However, its broad commercial prospects have long been constrained by the high cost, high energy consumption, and complex and environmentally detrimental traditional preparation process [3,5,6,7]. Most current mainstream processes rely on expensive organosilicon sources such as tetraethyl orthosilicate (TEOS) and require time-consuming solvent replacement and energy-intensive supercritical drying (SCD) to prepare them, which directly leads to high production costs [8,9,10]. Economic analysis indicates that raw materials (mainly silicon sources and organic solvents) and drying process energy consumption account for 48% and 44% of the total production cost of aerogels, respectively [11]. Therefore, developing a preparation strategy that is environmentally friendly, cost-effective, and simple to process is key to realizing the large-scale application of silica aerogels.

To achieve cost reduction and greening of raw materials, aqueous sodium silicate (commonly referred to as water glass), which is widely available and inexpensive, is considered an ideal alternative to organosilicon sources [12,13,14]. Unlike TEOS, which must rely on alcohol solvents for hydrolysis-condensation, water glass can be directly dissolved in the aqueous phase and can form a gel through simple ion exchange, thus avoiding the use of organic solvents as the source [15,16,17,18]. However, simply replacing the raw materials is not enough to achieve a green and simple overall process; the subsequent drying and hydrophobic modification steps remain the main bottlenecks. Although supercritical drying technology is relatively mature, its harsh high-pressure conditions bring high equipment investment and operating costs [19,20]. Although atmospheric pressure drying requires less demanding equipment, it still faces the challenge of skeletal collapse caused by capillary forces during drying [21,22]. For example, Pan et al. achieved the rapid preparation of water-glass-based silica aerogel powders via ambient pressure drying, demonstrating the feasibility of using low-cost silicate precursors [23]. However, the ambient pressure drying route employed still requires repeated solvent exchange and surface modification in an organic medium to mitigate capillary stress during drying, which increases solvent consumption, process complexity, and overall costs.

Vacuum freeze-drying technology offers a promising solution to the challenges. This technology utilizes the principle of ice crystal sublimation to directly remove moisture from the pores of the gel network, eliminating gas–liquid interfacial tension, preventing the collapse of the framework during the drying process, and is particularly suitable for water-based gel systems [14,24]. Although this method is environmentally friendly, the surface of the silica aerogel obtained by freeze-drying is rich in hydrophilic silanol groups, which easily absorb moisture from the environment, leading to structural degradation. Therefore, effective hydrophobic modification is necessary [25,26]. For example, Zhang et al. prepared hydrophobic silica aerogels using methyltrimethoxysilane (MTMS) and water glass as co-precursors, combined with freeze-drying [27]. Traditional liquid-phase modification methods are limited by the diffusion efficiency of reagents in nanopores and still rely on solvent replacement processes, not eliminating dependence on organic solvents [28,29,30]. In contrast, solvent-free vapor deposition technology allows for effective in-situ hydrophobic modification by directly reacting gaseous silane with the material surface. This avoids the large amount of organic waste generated by liquid-phase hydrophobic modification, making it an ideal choice for matching green drying processes [31,32].

Building on the above analysis, this study aims to develop a novel, environmentally friendly, and economical preparation strategy that integrates the entire process of raw material preparation, drying, and modification. In this study, low-cost water glass was employed as the silicon precursor, while deionized water served as the only solvent. The effects of temperature, concentration, pH, and catalyst concentration in the sol–gel process were systematically optimized using an orthogonal experimental design. The resulting wet gel was directly freeze-dried under vacuum, completely avoiding the traditional organic solvent replacement step, and in-situ hydrophobic modification of the prepared aerogel was performed using solvent-free vapor deposition. Meanwhile, the effectiveness and feasibility of the proposed integrated strategy were verified through a comprehensive investigation of the aerogel microstructure, pore characteristics, surface chemical composition, hydrophobicity, and thermal insulation behavior by means of scanning electron microscopy (SEM), Fourier-transform infrared spectroscopy (FTIR), nitrogen adsorption–desorption analysis (BET), and water contact angle measurements.

## 2. Experimental

### 2.1. Materials

Sodium silicate (Na_2_SiO_3_) was purchased from Jiashan Yourui Refractory Materials Co., Ltd. (Jiaxing, China); 732 cation exchange resin was obtained from Shanghai Yuanye Bio-Technology Co., Ltd. (Shanghai, China); glacial acetic acid (CH_3_COOH, 99%) was purchased from Shanghai Aladdin Biochemical Technology Co., Ltd. (Shanghai, China); aqueous ammonia (NH_3_·H_2_O, 99%) was purchased from Sinopharm Chemical Reagent Co., Ltd. (Shanghai, China); hexamethyldisilazane (HMDS, 99%) was obtained from Shanghai Macklin Biochemical Technology Co., Ltd. (Shanghai, China). Deionized water (H_2_O) was prepared using a laboratory ultrapure water system. All chemicals were of analytical grade and used as received without further purification.

### 2.2. Preparation of Aerogels

**Figure 1:**
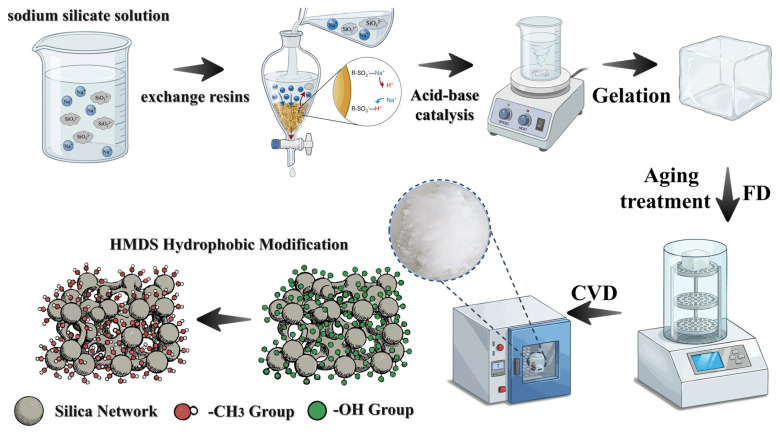
Experimental Flowchart.

First, sodium ions were removed from the precursor sodium silicate using 732 cation exchange resin, and the de-sodiated sodium silicate was diluted to the desired concentration. An appropriate amount of glacial acetic acid was added to the diluted sodium silicate and stirred at a constant temperature for 15 min to ensure complete hydrolysis of the precursor. Afterward, aqueous ammonia was introduced to regulate the pH and accelerate the sol–gel transformation process. The resulting wet gels were aged at 50 °C, allowing the gel framework to develop greater integrity and stability. After aging, the samples were immersed in deionized water for 24 h, with the washing solution replaced every 8 h to remove any remaining reactants and inorganic salt byproducts. Next, the cleaned wet gels were quickly frozen in liquid nitrogen to minimize shrinkage of the network during drying. They were then freeze-dried under vacuum to yield the hydrophilic silica aerogel structure. Finally, the hydrophilic silica aerogel was subjected to hydrophobic modification using HMDS vapor, yielding the hydrophobic silica aerogel sample (Figure 1).

### 2.3. Characterization

The surface morphology of the samples was examined using a scanning electron microscope (SEM, Quanta 200, Thermo Fisher Scientific, Hillsboro, OR, USA). Fourier-transform infrared (FTIR) spectra were recorded on a NEXUS 670 spectrometer (Nicolet, Madison, WI, USA) in the range of 500–4000 cm^−1^. Specific surface area and pore structure were analyzed using an automatic surface area and pore size analyzer (ASAP 2460, Micromeritics Instrument Corporation, Norcross, GA, USA). The specific surface area was determined from the adsorption data, and the pore size distribution and pore volume were calculated using the BJH method. Water contact angles of the samples were measured with a contact angle goniometer (Dataphysics OCA 20, Dataphysics Instruments GmbH, Filderstadt, Germany). Thermogravimetric–differential scanning calorimetry (TG-DSC) analyses were conducted using a simultaneous thermal analyzer (NETZSCH STA 449 F5, NETZSCH-Gerätebau GmbH, Selb, Germany) under an argon atmosphere from room temperature to 800 °C. Thermal conductivity was measured using the transient plane source method (Hot Disk TPS2500S, Hot Disk AB, Göteborg, Sweden). The porosity of the samples was calculated according to the following equation [15]:

(1) $$porosity=\left(1-\frac{\rho_{a}}{\rho_{s}}\right)\times 100\%$$

where *ρ_a_* is the apparent density of the aerogel and *ρ_s_* is the skeletal density of the silica framework. Since the aerogel skeleton is mainly composed of amorphous SiO_2_, *ρ_s_* was taken as 2.2 g/cm^3^, which is a commonly reported density for amorphous silica [16].

## 3. Results and Discussion

### 3.1. Orthogonal Optimization of Gelation Process Parameters

In a fully aqueous system, the formation of the aerogel’s mesoporous network is largely governed by the hydrolysis kinetics and condensation dynamics of the primary silica particles [15]. Therefore, compared with conventional auxiliary processes such as aging or washing, the precursor concentration, system temperature and the amount of acid–base catalysts are the key factors affecting porosity and skeleton strength [17]. To comprehensively investigate how these factors affect the structure and properties of silica aerogels derived from water glass, this study used reaction temperature, water glass concentration, CH_3_COOH concentration and NH_3_·H_2_O concentration as experimental factors, and designed 9 sets of experiments based on an L9(3^4^) orthogonal experimental scheme, with the corresponding sample groups listed in Table 1 and denoted as SA-1 to SA-9. Meanwhile, yield was selected as one evaluation index, while pore characteristics and thermal insulation performance were used as additional assessment parameters, as summarized in Table 2. The average response value (*K_i_*) and range value (*R*) for each factor at different levels were determined using Equations (2) and (3), respectively:

(2) $$K_{i}=\frac{\sum X_{i}}{3}$$

(3) $$R=max\left(k_{i}\right)-min\left(k_{i}\right)$$

In the formula, *X_i_* represents the experimental measurement result of yield, specific surface area, or thermal conductivity.

Table 3 shows the range analysis results of the orthogonal experiment. Based on the *K_i_* and *R* values at different levels of each factor, the effects of various process parameters on the yield, BET, and thermal conductivity of silica aerogels can be evaluated. *K_i_* is used to determine the optimal process level for each factor. The *R* value indicates the influence of the factor on the results.

**Figure 2:**
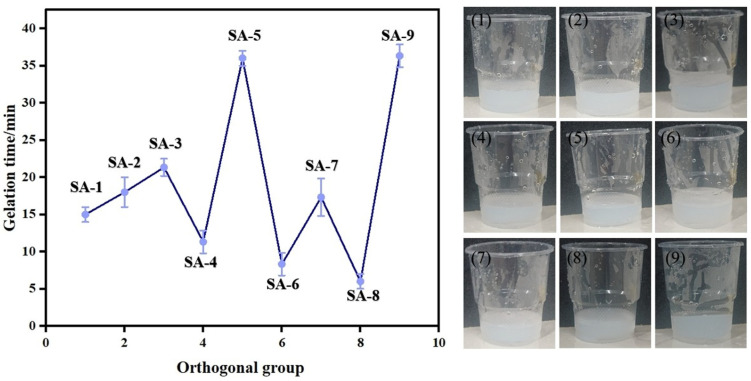
Gel Time and Gel State Diagram of 9 Orthogonal Experiments.

As shown in Figure 2, all nine samples prepared using an orthogonal experimental design successfully underwent the sol–gel reaction and formed gels. Meanwhile, the gelation times of the nine samples ranged from 6 to 36 min, exhibiting noticeable kinetic differences. This indicates that the differences in process conditions within the orthogonal groups significantly modulated the condensation rate of the silicic acid monomers. For example, sample SA-8 had a gelation time of only 6.0 min, while SA-9 had a gelation time of 36.6 min. This is because during the sol–gel transition in SA-8, the environment of silanol monomers was more alkaline, thereby accelerating the condensation process and facilitating the rapid construction of the network structure. In contrast SA-9 is produced in a less alkaline environment for silanol monomers during the gelation process, resulting in a relatively lower condensation reaction rate and a delayed gelation process. Therefore, the gelation time needs to be controlled within an appropriate range, as it reflects whether the silanol condensation rate in the system is within a reasonable range.

The yield of the aerogel is directly related to the production cost and scalability potential of this green preparation process. The range analysis indicates that reaction temperature plays a dominant role in controlling aerogel yield, as it influences the final output by regulating the sol–gel reaction kinetics. As presented in Table 3, the aerogel yield exhibits an obvious upward trend with increasing reaction temperature. Specifically, when T rises from 25 °C (A_3_) to 35 °C (A_1_), a significant improvement in yield can be observed. This surge in yield is directly controlled by sol–gel kinetics. There is an activation energy barrier in the condensation process of silica monomers. The higher reaction temperature (35 °C) effectively intensifies the molecular thermal motion and collision frequency, which promotes the rapid condensation and solidification of the silicon–oxygen framework in the early stage of gelation [17]. This helps to enhance the network structure of the wet gel, thereby effectively preventing the loss of incompletely condensed oligomers in subsequent experimental processes, which is conducive to improving the yield and facilitating rapid preparation while maintaining product quality. In addition, aqueous ammonia is a key alkaline catalyst for controlling the condensation reaction rate. Its amount has the second greatest impact on yield after the reaction temperature. This is because the condensation activation energy between silanol groups decreases in an alkaline environment, leading to more rapid nucleation and growth of silica particles [33].

According to the range analysis presented in Table 3, the acid dosage (C, *R* = 318.74) has the most significant effect on the specific surface area of the aerogel among all investigated factors. As the acid dosage increases, the value rises significantly from 618.9584 m^2^/g at C_1_ to 824.6812 m^2^/g at C_3_. This result can be attributed to the regulation of sol–gel reaction kinetics under different process conditions [18,34]. The increased acid amount for C_3_ promotes the hydrolysis of water glass-derived silica precursor, while under the given alkaline catalysis conditions, the condensation reaction rate is relatively limited, thus achieving a more moderate kinetic equilibrium between hydrolysis and condensation processes. Subsequently, the finely dispersed primary particles assemble during the condensation process to form a network architecture with an increased surface area.

**Figure 3:**
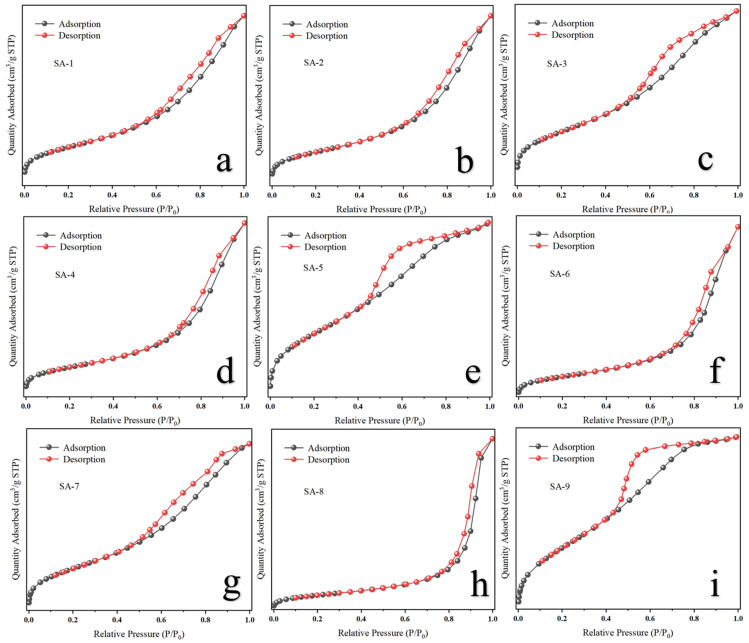
N_2_ adsorption-desorption isotherms of 9 groups of samples (**a**–**i**).

Figure 3 presents N_2_ adsorption–desorption isotherms that further verify the structural advantages of the all-aqueous system. All samples exhibited type IV isotherm profiles, indicating the successful formation of a well-developed mesoporous structure within the aqueous system. Under suitable acid–base ratios, SA-5 (C_3_) exhibited a typical H1 type hysteresis loop with a pore size highly concentrated at 3.3713 nm and a specific surface area as high as 858.8500 m^2^/g. This refined pore structure originates from the well-matched reaction kinetics in the aqueous system, achieving perfect synergy between acid catalyst-induced hydrolysis to generate monomers and base catalyst-induced condensation to form the network, allowing primary particles to grow uniformly. In contrast, the sample SA-8 (C_1_) prepared under insufficient acid or excessively alkaline conditions exhibited an isotherm that evolved into type H3, accompanied by a noticeable broadening of pore sizes and a significant reduction in surface area to 511.2497 m^2^/g. This indicates that the mesoporous network structure can be effectively tuned into the aqueous system by controlling the catalytic conditions. Furthermore, TEOS-derived silica aerogels generally exhibit specific surface areas of 900–1200 m^2^/g and pore diameters of approximately 8–10 nm; however, their preparation typically involves alcohol-based reaction media, solvent exchange, and liquid-phase surface modification [17,18,19,20]. Although the specific surface area of SA-5 (858.85 m^2^/g) is slightly lower than the highest values reported for some TEOS-derived aerogels, its type IV adsorption isotherm, H1-type hysteresis loop, and mesopore distribution confirm the formation of a well-developed mesoporous network. Therefore, the primary advantage of the present system lies not simply in achieving a higher specific surface area, but in maintaining competitive textural properties while using inexpensive water glass and an aqueous reaction medium, thereby reducing reliance on costly alkoxide precursors and organic solvents.

**Figure 4:**
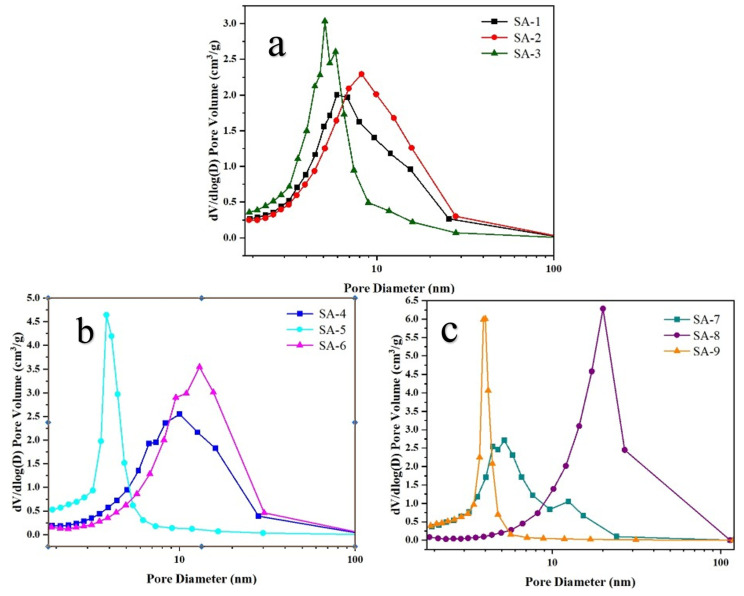
Pore size distribution diagrams of 9 orthogonal experimental samples (**a**–**c**).

Thermal conductivity serves as an important criterion for evaluating the thermal insulation capability of aerogels [35]. Table 3 shows that the effects of different process parameters on aerogel thermal conductivity are relatively limited, the range *R* value of each factor changes very little, and the thermal conductivity is stable at around 0.040. Among all samples, SA-5, which has the highest specific surface area, also presents a value of 0.040 W/(m·K). This behavior is closely related to the pore size distribution shown in Figure 4. Although the samples exhibit different pore dimensions under varying conditions, ranging from 3.3713 nm for SA-5 to 13.9266 nm for SA-8, all values fall within the typical mesoporous range. Since these pores are much smaller than the mean free path of air molecules at room temperature (approximately 70 nm), gas-phase heat transfer is predominantly controlled by collisions between gas molecules and pore walls, thereby effectively suppressing gaseous thermal conduction [36]. Moreover, the complex condensed nano framework structure inside the aerogel significantly reduces the solid phase heat conduction efficiency. This contributes to its excellent and stable thermal insulation performance. These results demonstrate the positive effect of the fine skeletal structure on suppressing gas-phase heat conduction.

To balance the microstructure, preparation efficiency, and thermal insulation performance of the aerogel, this study comprehensively evaluated the results of orthogonal experiments. Compared to samples with outstanding single properties, the A_1_B_1_C_2_D_2_ combination achieved a higher yield while maintaining a high specific surface area and low thermal conductivity and was therefore established as the optimal process scheme. The results of the three parallel validation experiments summarized in Table 4 demonstrate that the silica aerogel achieves an average yield (Q) of 0.9095 g, a specific surface area (S) of 707.8773 m^2^/g, a thermal conductivity (K) of 0.0408 W/(m·K), an average pore diameter (P) of 5.7572 nm, a density of 0.0245 g/cm^3^, a porosity of 98.89%, and a gelation time of approximately 20 min. Compared with traditional synthesis systems, the aqueous system exhibits clear advantages in three main aspects: First, it avoids the use of organic solvents, eliminating the cumbersome processing steps of solvent replacement and recovery, and greatly simplifying the preparation process. Second, it has high reactivity, which is beneficial for rapid preparation. Third, the resulting aerogel possesses excellent structural characteristics of high porosity and narrow pore size distribution, laying a structural foundation for subsequent hydrophobic modification and thermal insulation applications.

### 3.2. Chemical Vapor Phase Hydrophobic Modification and Performance Regulation

Based on the optimal synthesis process (A_1_B_1_C_2_D_2_) identified by orthogonal experiments, using the amount of HMDS as the single variable (amounts of 0, 0.5, 1.0, 1.5 and 2.0 mL, respectively), corresponding to numbers S0–S4, the effects of vapor-phase modification on the surface chemistry, wettability, and microstructural characteristics of the aerogel were systematically investigated.

#### 3.2.1. Evolution of Surface Chemical Structure

**Figure 5:**
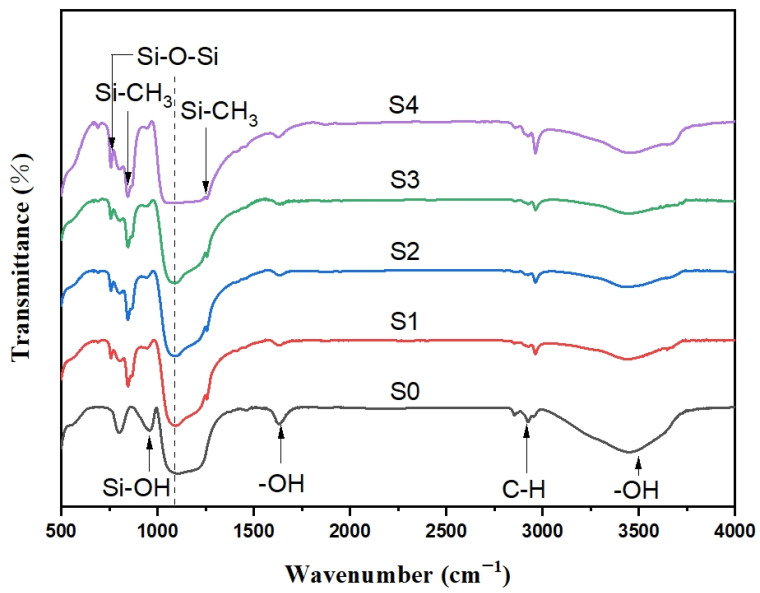
Infrared spectra of S0–S4 aerogel samples.

The FTIR spectra in Figure 5 reveal the vibration of characteristic peaks during the modification of aerogel by HMDS. All samples showed characteristic Si-O-Si vibration peaks at 1045 cm^−1^ and 776 cm^−1^, verifying the successful formation of the silica network structure [37]. The unmodified S0 sample showed O-H stretching vibrations at 3450 cm^−1^ and Si-OH stretching vibrations at 953 cm^−1^, indicating that the surface is rich in hydrophilic hydroxyl groups [20]. From sample S1 to S4, as the amount of modifier increased, the peak intensities of Si-CH_3_ at 1275 cm^−1^ and 852 cm^−1^ and C-H at 2966 cm^−1^ gradually increased, while the peak intensity of -OH at 3450 cm^−1^ decreased synchronously, indicating that the surface silanol groups were gradually replaced and hydrophobic methyl groups were successfully introduced. This process is essentially a stepwise silylation reaction between HMDS and surface Si-OH, releasing byproducts such as NH_3_ during the reaction and forming a stable Si-O-Si (CH_3_)_3_ structure on the surface [3]. Furthermore, the aerogel prepared by the aqueous system has a relatively uniform distribution of silanol groups on its surface, which provides more sufficient reaction sites for HMDS, thus facilitating more uniform vapor-phase hydrophobic modification.

#### 3.2.2. Analysis of Surface Wettability

**Figure 6:**
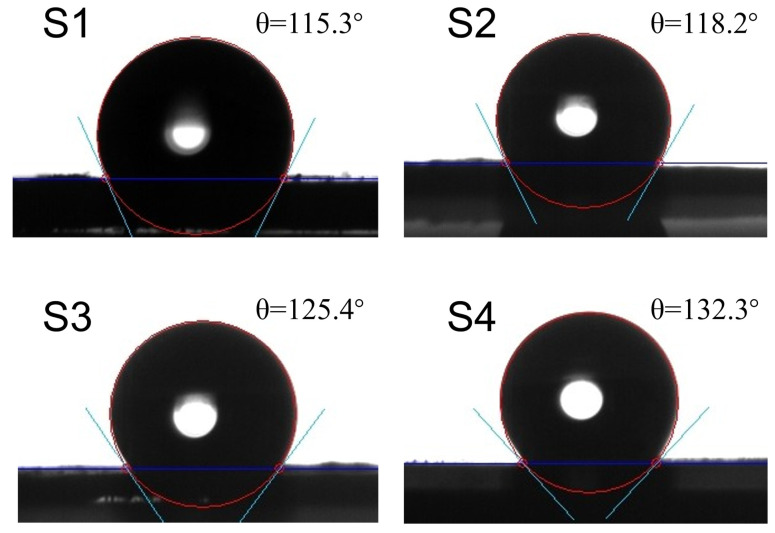
Hydrophobic angle testing of S1–S4 aerogel samples.

Figure 6 illustrates that the water contact angles of samples S1–S4 steadily rose from 115.3° to 132.3°, demonstrating a significant enhancement in hydrophobicity. Furthermore, the contact angles increased with increasing modifier dosage, indicating successful hydrophobic modification of the aerogel surface. This change is in good agreement with the trend of increasing Si-CH_3_ characteristic peak intensity and gradually decreasing Si-OH characteristic peak intensity in FTIR. This phenomenon primarily arises from the reduction in surface free energy and the altered interaction with water molecules following the silylation of silanol groups, thus achieving a hydrophobic transition. The high contact angle of 132.3° for sample S4 further demonstrates that vapor deposition enables in-situ hydrophobic modification without the need for organic solvents, thereby enabling controlled tuning of the hydrophobic properties of the aerogel.

#### 3.2.3. Analysis of BET

**Figure 7:**
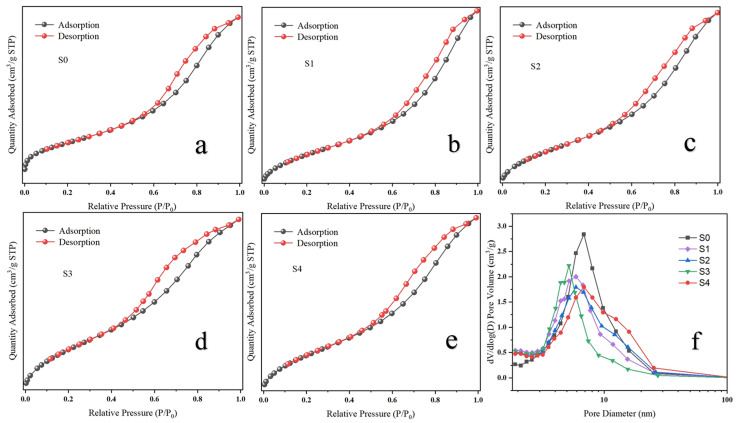
Adsorption–desorption isotherms of aerogel samples ((**a**–**e**): S0–S4), pore size distribution of aerogel samples ((**f**): S0–S4).

The N_2_ adsorption–desorption isotherms presented in Figure 7, along with the BET data in Table 5, illustrate how gas-phase modification influences the aerogel pore characteristics, which can be interpreted in two distinct stages. Initially, the S0 sample exhibits a type IV isotherm, with a surface area of 706.64 m^2^/g and an average pore diameter of 5.76 nm. As the HMDS content increases to S3, the surface area decreases to 488.93 m^2^/g, adsorption at low pressures diminishes, and the pore distribution gradually shifts toward larger sizes. Moving from S3 to S4, additional HMDS leads to pronounced adsorption in the high-pressure region (P/P_0_ > 0.8), indicating enhanced capillary condensation. At the same time, the mean pore diameter rises to 8.73 nm, accompanied by broader pore size distribution, while the surface area remains nearly constant. This behavior results from the incorporation of -Si(CH_3_)_3_ groups, which occupy pore volume and modify the pore architecture, causing slight reductions in surface area and structural adjustments [38]. The results indicate that while HMDS vapor-phase modification imparts hydrophobicity to aerogels, it also has a certain impact on their pore structure, suggesting that the amount of modifier needs to be balanced between hydrophobicity and maintaining the pore structure.

#### 3.2.4. SEM Analysis

**Figure 8:**
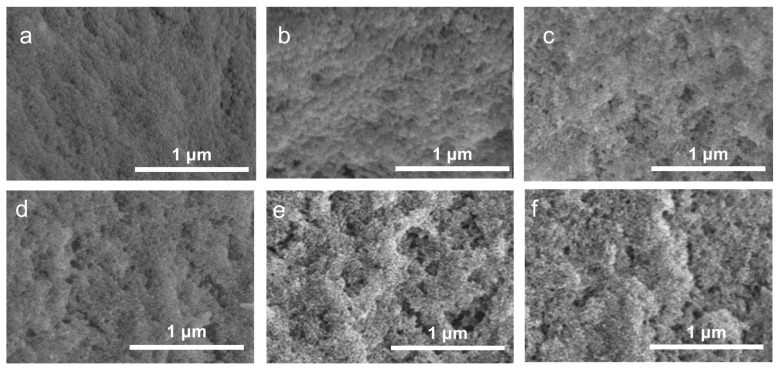
SEM micrographs of S0–S4 aerogel samples: (**a**) = S0; (**b**) = S1; (**c**) = S2; (**d**) = S3; (**e**,**f**) = S4.

Figure 8 illustrates the evolution of the microstructure of the S0–S4 aerogels. The unmodified S0 sample exhibits a relatively dense three-dimensional network composed of interconnected silica nanoparticles. Following HMDS modification, the particle boundaries become increasingly distinct, and the network structure takes on a more open and coarsened morphology. BET results provide quantitative support for this observation: the specific surface area decreases from 706.64 m^2^/g for S0 to 488.93 m^2^/g for S3, subsequently remaining essentially stable at 489.79 m^2^/g for S4; meanwhile, the average pore size shifts from 5.76 nm (S0) to 4.49 nm (S3) before increasing significantly to 8.73 nm (S4). These changes indicate that excessive HMDS modification promotes structural coarsening and pore enlargement, likely due to the grafting of bulky -Si(CH_3_)_3_ groups and the resulting rearrangement of the silica network [39]. Notably, the silica aerogels prepared in an aqueous system possess a well-ordered initial framework with high uniformity, ensuring the smooth progression of the hydrophobic modification reaction; furthermore, the aerogels retain an intact and stable framework following the modification treatment.

The modulation of silica aerogels by HMDS vapor deposition is a typical example of surface silylation modification. FTIR results show that the characteristic Si-OH peak is weakened while the Si-CH_3_ and C-H peaks are enhanced, indicating that HMDS reacts with the surface -OH groups and introduces hydrophobic groups. This process occurs in the dried silica (Si-O-Si) framework. Although the silica (Si-O-Si) framework remains largely unchanged, the interaction of hydrogen bonds between particles is likely weakened. SEM images show that the overall morphology of the silica aerogel sample remains unchanged, but the interfaces are clearer and the particle boundaries become more distinguishable. BET results indicate that the specific surface area initially decreases and subsequently increases with the HMDS content. This trend is attributed to the partial occupation of pore space and redistribution of the pore structure caused by grafted -Si(CH_3_)_3_ groups. Contact angle measurements further confirm a marked enhancement in the hydrophobicity of the modified aerogel. In summary, surface modification of silica aerogel using HMDS vapor deposition can optimize the pore structure and improve hydrophobicity without significantly altering the original silica framework structure. Moreover, this modification process is simple to operate, environmentally friendly, and low in cost, demonstrating significant comprehensive advantages.

### 3.3. Thermal Stability and Thermal Insulation Performance of the Aerogels

**Figure 9:**
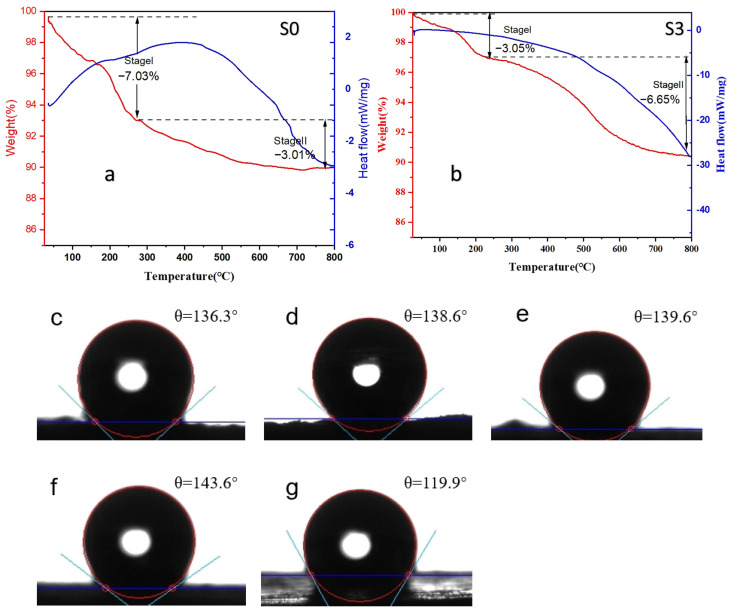
TG-DSC curves of S0 and S3 aerogel samples: (**a**) = S0; (**b**) = S3. Water contact angles of the HMDS-modified aerogel after heat treatment at different temperatures for 1 h: (**c**) 100 °C, (**d**) 150 °C, (**e**) 200 °C, (**f**) 250 °C, and (**g**) 300 °C.

Figure 9 presents the thermogravimetric profiles of the aerogel before and after vapor deposition, highlighting how surface modification influences its composition and thermal stability. For the unmodified sample S0, a mass loss of 7.03% occurs from room temperature to 280 °C, and the DSC curve shows an endothermic trend. This is mainly attributed to the rapid evaporation of a large amount of physically adsorbed water in the aerogel network, reflecting the hydrophilic characteristics of the material [40]. At 280–800 °C, a mass loss of 3.01% occurs which can be attributed to the high-temperature dehydration condensation of residual silanol (Si-OH) groups within the material [41]. Compared with sample S3, the mass loss in the first stage was only 3.05%. Grafted-Si(CH_3_)_3_ groups effectively prevent water molecules from adhering to the aerogel surface, resulting in superior hydrophobic performance. At 280–800 °C, the mass loss of S3 increases significantly to 6.65%, primarily attributed to the thermal decomposition of the grafted organic groups [42]. Existing studies indicate that HMDS-modified silica aerogels generally remain stable up to approximately 267–300 °C, whereas surface methyl groups gradually decompose over a broader temperature range, with the specific decomposition behavior influenced by the test atmosphere [43,44]. The second stage of weight loss observed above approximately 280 °C in this study is broadly consistent with findings reported in the literature. The decomposition of organic groups occurs continuously and gradually over a wide temperature range. Therefore, the DSC curves in this range show only a gentle heat flow drift without sharp thermal peaks, indicating that the modified aerogel maintains good structural stability at elevated temperatures.

Given the material stability requirements for potential industrial applications, the stability of the water contact angle for sample S3 was further investigated across various heat treatment temperatures. Sample S3 was subjected to heat treatment at 100 °C, 150 °C, 200 °C, 250 °C, and 300 °C for 1 h, followed by cooling to room temperature and measurement of the water contact angle. The water contact angle of the untreated S3 sample at room temperature was 125.4°. As shown in Figure 9, after heat treatment at 100 °C, 150 °C, 200 °C and 250 °C, the water contact angles increased to 136.3°, 138.6°, 139.6°, and 143.6°, respectively; this indicates that moderate heat treatment can further enhance the material’s hydrophobicity, demonstrating good thermal stability. This phenomenon may be attributed to the removal of physisorbed water during heat treatment and the behavior of surface silanol groups—which are closely linked to water adsorption/desorption and thermal dehydroxylation processes—whereby a reduction in silanol groups lowers surface hygroscopicity and enhances hydrophobic characteristics [45]. When the treatment temperature was raised to 300 °C, the contact angle dropped to 119.9°—lower than the 125.4° observed for the untreated sample—likely due to the partial decomposition of methyl-containing hydrophobic groups and the exposure of hydrophilic sites at high temperatures [38]. Nevertheless, the contact angle remained above 90° after treatment at 300 °C, indicating that the aerogel retained a degree of hydrophobicity even after high-temperature treatment.

**Figure 10:**
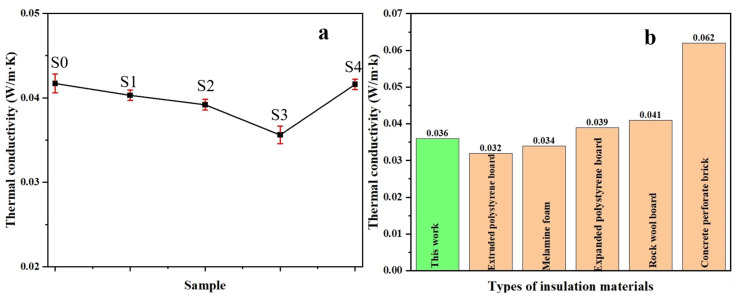
Thermal conductivity variation of Si-based aerogels S0–S4 (**a**); thermal conductivity of common building insulation materials (**b**).

Thermal conductivity is a key parameter for evaluating the heat-insulating performance of aerogels, as it reflects their efficiency in impeding heat transfer. Figure 10 shows the thermal conductivity of the aerogel before and after modification. The data in (a) are based on three independent measurements and are presented as average and error. The results indicate that proper hydrophobic modification markedly enhances the aerogel’s thermal insulation. Figure 10a illustrates that the thermal conductivity of the aerogel decreases at first and then rises as HMDS content increases, with sample S3 reaching the lowest value of 0.0360 W/(m·K). The excellent thermal insulation of the aerogel is mainly due to its nanoscale pore structure, which limits multiple modes of heat transfer. Notably, the average pore size of S3 is 4.49 nm, much smaller than the mean free path of air molecules (~70 nm) at ambient conditions, thereby effectively hindering gas-phase thermal conduction [13]. In addition, the complex cross-linked nano framework and dense pore walls not only greatly extend the solid phase heat conduction path but also reduce the heat radiation transfer [46]. Based on the comparison of thermal conductivities in Figure 10b, it is evident that the aerogel prepared in this work outperforms conventional commercial insulation materials in overall performance. Currently, commercial thermal insulation materials are primarily divided into two categories. One is organic foam materials that rely on petrochemical resources, such as extruded polystyrene foam board XPS and melamine foam, and the other is inorganic thermal insulation materials that are prepared with high energy consumption, such as rock wool. In comparison, the hydrophobic silica aerogel prepared in this study is based on inexpensive and abundant water glass, with water serving as the sole solvent in the sol–gel process. In this aqueous route, water replaces conventional organic solvents as the reaction medium, offering advantages in terms of environmental compatibility, safety, and cost. The use of freeze-drying further avoids the high-pressure conditions required for supercritical drying, making the process simpler and more suitable for scale-up. Therefore, this method provides a greener, lower-cost, and potentially scalable strategy for preparing hydrophobic silica aerogels.

## 4. Conclusions

This study demonstrates the preparation of hydrophobic silica aerogels using low-cost water glass as the raw material, combining orthogonal optimization, freeze-drying, and vapor-phase deposition. A highly porous mesoporous structure was established by tuning the parameters of the sol–gel process, while vapor-phase treatment with HMDS transformed the material surface from hydrophilic to hydrophobic without compromising the integrity of the silica framework. The modified S3 sample exhibited a water contact angle of 132.3° and a thermal conductivity of 0.0360 W/(m·K), demonstrating a favorable balance among pore structure, surface hydrophobicity, and thermal insulation performance. Crucially, this route employs water as the reaction medium—thereby avoiding extensive organic solvent exchange—and utilizes freeze-drying instead of high-pressure supercritical drying; this reduces reliance on expensive alkoxide silica sources and liquid organic solvents, offering a novel approach for the green and cost-effective production of silica aerogels. Future research could focus on long-term hygrothermal stability and process scale-up to provide insights for the practical application of water glass-based thermal insulation aerogels.

## Figures and Tables

**Table 1:** Four factors and three levels of the 9 groups of the aerogel sample array.

Level i	Factors			
	A Temperature	B WG/H_2_O	C CH_3_COOH/WG	D NH_3_·H_2_O/WG
1	35 °C	1.05 × 10^−2^	2.1	1.4
2	30 °C	1.35 × 10^−2^	2.3	1.6
3	25 °C	1.65 × 10^−2^	2.5	1.8

**Table 2:** The orthogonal experimental array with test results of 9 groups of samples.

Exp. Number	Temperature	WG/H_2_O	Hac/WG	NH_3_·H_2_O/WG	Q g	S m^2^/g	P nm	K W/(m·K)
SA-1	35 °C	1.05 × 10^−2^	2.1	1.4	0.8077 ± 0.0023	696.6055	6.2976	0.0416
SA-2	35 °C	1.35 × 10^−2^	2.3	1.6	0.9011 ± 0.0015	733.6092	7.0101	0.0417
SA-3	35 °C	1.65 × 10^−2^	2.5	1.8	0.6670 ± 0.0013	759.0155	4.7532	0.0423
SA-4	30 °C	1.05 × 10^−2^	2.3	1.8	0.6478 ± 0.0046	681.4718	7.9794	0.0411
SA-5	30 °C	1.35 × 10^−2^	2.5	1.4	0.7112 ± 0.0065	858.8500	3.3713	0.0400
SA-6	30 °C	1.65 × 10^−2^	2.1	1.6	0.6268 ± 0.0031	649.0199	9.5399	0.0418
SA-7	25 °C	1.05 × 10^−2^	2.5	1.6	0.7298 ± 0.0038	856.1782	5.0866	0.0413
SA-8	25 °C	1.35 × 10^−2^	2.1	1.8	0.3915 ± 0.0051	511.2497	13.9266	0.0436
SA-9	25 °C	1.65 × 10^−2^	2.3	1.4	0.5912 ± 0.0023	626.9448	3.5152	0.0434

Note: Q is the sample yield, S represents the specific surface area of the sample, P represents the average pore diameter of the sample determined by the BJH method, and K is the thermal conductivity.

**Table 3:** Range analysis of orthogonal experiments for yield, specific surface area and thermal conductivity.

Exp. Number	A Temperature	B WG/H_2_O	C CH_3_COOH/WG	D NH_3_·H_2_O/WG	
Average ρ = 1 (K_1_)	729.7434	744.7518	618.9584	727.4668	surface area
Average ρ = 2 (K_2_)	729.7806	701.2363	680.6753	746.2691	
Average ρ = 3 (K_3_)	664.7909	678.3267	824.6812	650.5790	
Range (R_j_)	64.9896	66.4251	205.7229	95.6901	
S of factors		C > D > B > A			
Average ρ = 1 (K_1_)	0.0418	0.0413	0.0423	0.0416	thermal conductivity
Average ρ = 2 (K_2_)	0.0409	0.0417	0.0420	0.0416	
Average ρ = 3 (K_3_)	0.0427	0.0425	0.0412	0.0423	
Range (R_j_)	0.0018	0.0012	0.0011	0.0007	
K of factors		A > B > C > D			
Average ρ = 1 (K_1_)	0.7919	0.7284	0.6086	0.7033	quality output
Average ρ = 2 (K_2_)	0.6619	0.6679	0.7133	0.7525	
Average ρ = 3 (K_3_)	0.5708	0.6283	0.7026	0.5687	
Range (R_j_)	0.2211	0.1001	0.1047	0.1838	
Q of factors		A > D > C > B			

**Table 4:** A_1_B_1_C_2_D_2_ group silicon-based aerogel validation experiment results.

	Q g	P nm	S m^2^/g	K W/(m·K)	Gelation Time Min
VE1	0.9106	5.7608	706.8459	0.0417	20
VE2	0.9086	5.6985	697.3241	0.0399	21
VE3	0.9092	5.8124	719.4618	0.0407	20
$\bar{X}$	0.9095	5.7572	707.8773	0.0408	20.3333

**Table 5:** Specific Surface Area and Pore Size of S0-S4 Aerogel Samples.

	S0	S1	S2	S3	S4
S m^2^/g	706.6409	536.6275	501.8966	488.9327	489.7882
P nm	5.7608	4.9650	5.3880	4.4929	8.7279

## Data Availability

The original contributions presented in this study are included in the article. Further inquiries can be directed to the corresponding author.
